# Supplementary figures and images for: Unveiling the Catalytic Roles of DsBBS1 and DsBBS2 in the Bibenzyl Biosynthesis of Dendrobium sinense
Source: Molecules. 2024 Aug 3;29(15):3682. doi: 10.3390/molecules29153682 (PMC11314366; doi:10.3390/molecules29153682)

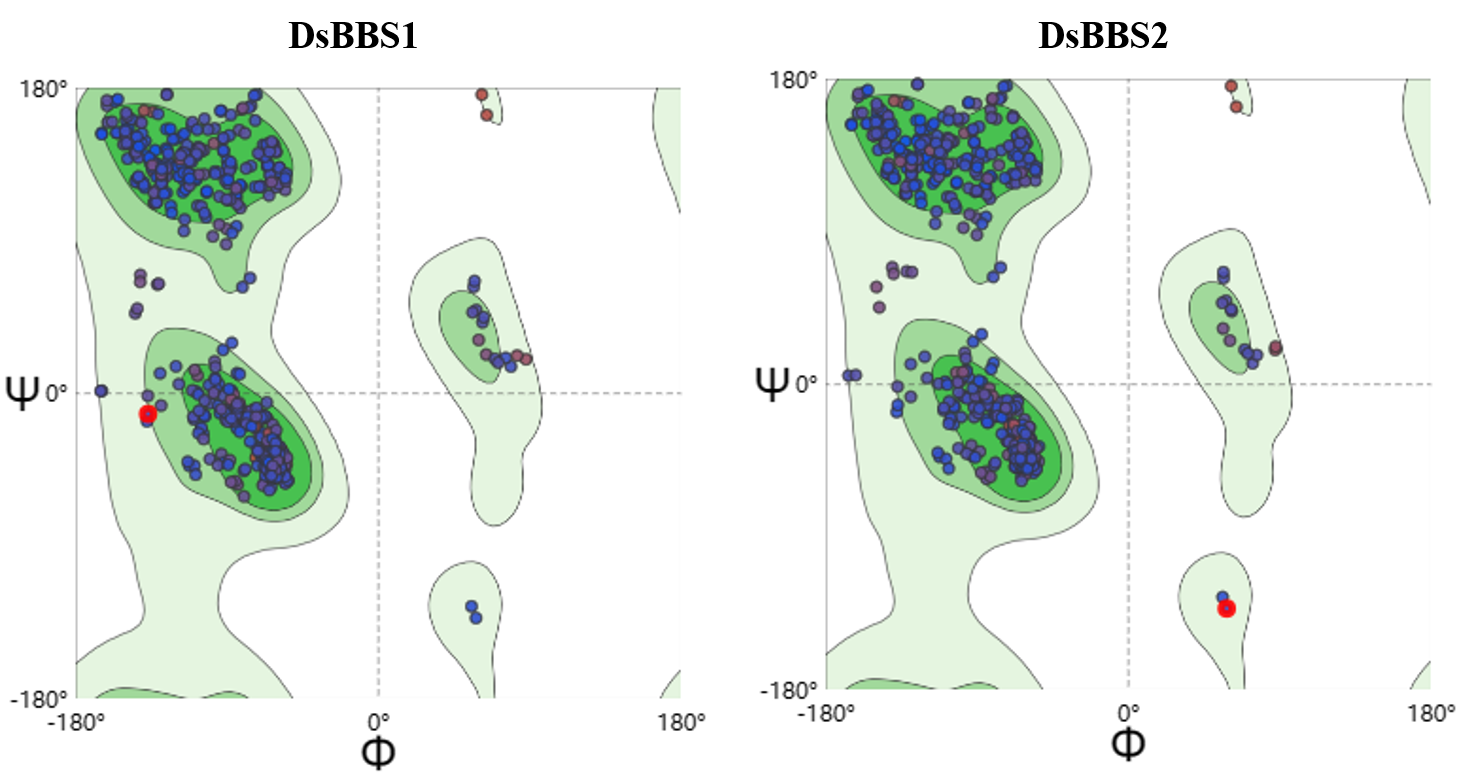

Supplement: Supplementary file 1 [file molecules-29-03682-s001.zip › Figure S1.png]

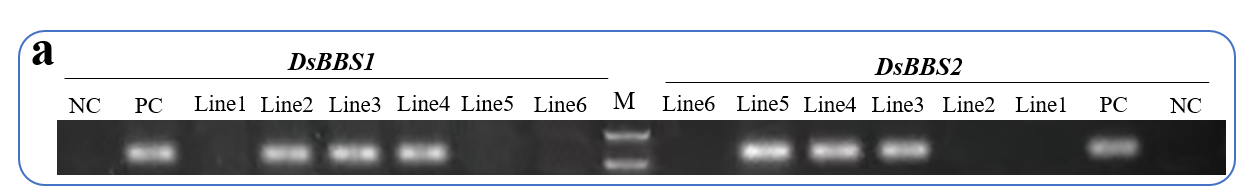

Supplement: Supplementary file 1 [file molecules-29-03682-s001.zip › Figure S2.png]
